# Glioblastoma lacking necrosis or vascular proliferations: Different clinical presentation but similar outcome, regardless of histology or isolated *TERT* promoter mutation

**DOI:** 10.1093/noajnl/vdad075

**Published:** 2023-06-21

**Authors:** Maarten M J Wijnenga, Sybren L N Maas, Vera van Dis, C Mircea S Tesileanu, Johan M Kros, Linda Dirven, Hans M Hazelbag, Hendrikus J Dubbink, Arnaud J P E Vincent, Pim J French, Martin J van den Bent

**Affiliations:** Department of Neurology, Brain Tumor Center, Erasmus MC Cancer Institute, Rotterdam, The Netherlands; Department of Pathology, Brain Tumor Center, Erasmus MC Cancer Institute, Rotterdam, The Netherlands; Department of Pathology, Leiden University Medical Center, Leiden, The Netherlands; Department of Pathology, Brain Tumor Center, Erasmus MC Cancer Institute, Rotterdam, The Netherlands; Department of Neurology, Brain Tumor Center, Erasmus MC Cancer Institute, Rotterdam, The Netherlands; Department of Pathology, Brain Tumor Center, Erasmus MC Cancer Institute, Rotterdam, The Netherlands; Department of Neurology, Leiden University Medical Center, Leiden, The Netherlands; Department of Pathology, Medical Center Haaglanden, The Hague, The Netherlands; Department of Pathology, Brain Tumor Center, Erasmus MC Cancer Institute, Rotterdam, The Netherlands; Department of Neurosurgery, Brain Tumor Center, Erasmus MC Cancer Institute, Rotterdam, The Netherlands; Department of Neurology, Brain Tumor Center, Erasmus MC Cancer Institute, Rotterdam, The Netherlands; Department of Neurology, Brain Tumor Center, Erasmus MC Cancer Institute, Rotterdam, The Netherlands

In the 2021 revised World Health Organization Classification of Central Nervous System Tumours (WHO CNS5) classification, isocitrate dehydrogenase 1/2 -wild-type (IDHwt) gliomas lacking necrosis and/or vascular proliferations, but with a *TERT*-promotor (*TERT*p) mutation, and/or *EGFR* amplification, and/or combined gain of chromosome 7 and loss of chromosome 10 (7+/10−), are now classified as IDHwt glioblastoma.^1^ These formerly labeled “diffuse astrocytomas, IDH wild-type, with genetic features of glioblastoma” are not separated anymore from histologically defined glioblastomas. However, there is still discussion if these gliomas are truly the same in terms of first presentation and survival.^2^ In 2021, a French series concluded that glioblastoma patients with histology lacking high-grade features have a favorable outcome, with a median overall survival (OS) of 88 months in cases lacking anaplasia, increased mitotic activity, necrosis, or vascular proliferations when only a *TERT*p mutation is present.^3^ This challenges the concept of the proposed CNS5 classification of glioblastoma. Therefore, we expanded and re-analyzed a previously published cohort of 71 glioblastoma patients that lacked high-grade features in the original histopathological diagnosis and also showed imaging features that are more compatible with a lower-grade glioma.^4,5^ To investigate if histological grading had impact on OS in glioblastoma, we reevaluated the histological grade by 2 independent reviewers. We assessed OS for the different histological grades and also specifically investigated the OS impact of isolated *TERT*p mutations.

We identified 132 cases from 3 Dutch hospitals, which were initially histologically classified as grade 2 or 3 gliomas, but molecularly upgraded to GBM. Cases with ring-like contrast enhancement and/or central necrosis at time of diagnostic surgery were excluded, as a low-grade histology would then suggest the infiltration border was sampled and not the true histological appearance of the tumor. Two dedicated neuropathologists (SLNM/VD) blinded for clinical information independently revised the histological grade. Discordant cases were discussed to reach consensus. Out of 132 cases, tissue was available for revisions in 102 cases. Criteria for histology, analog to grade 3 in the 2016 WHO classification criteria, were anaplasia as defined by high cell density, nuclear polymorphism and/or nuclear hyperchromasia as well as increased mitotic activity. Detection of necrosis and/or endothelial proliferation warranted a grade 4. Patients were molecularly classified with a tailored NGS panel as described before.^4^ We compared OS in this cohort with OS of a historical histologically defined glioblastoma cohort (197 patients with MRI characteristics of glioblastoma (GBM) and GBM histology), of which characteristics were described before.^4^ For readability and clarity, we refer to “m-GBM“ for the glioblastoma cases lacking high-grade features, and “h-GBM” for the historical cohort in further text. Depending on each institution’s policy, the study received approval for exemption from review or was waived from review by the institution and was conducted according to national and local regulations.

**Figure 1.**
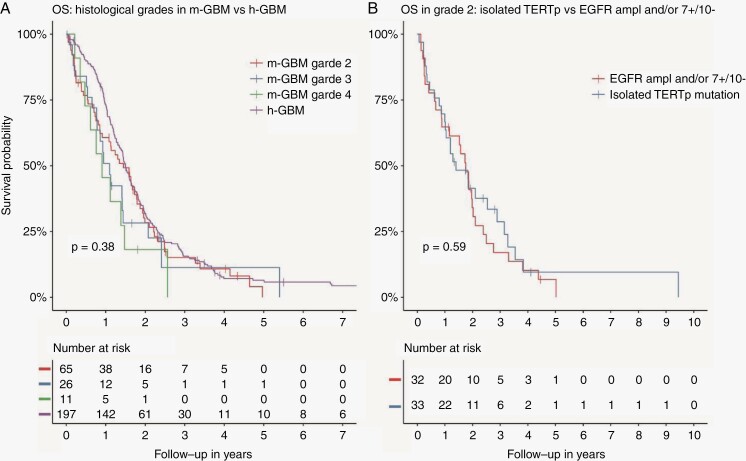
Panel A: overall survival curves for histological grades in m-GBM and the h-GBM cohort. Survival was defined as time from first surgery till date of death or censorship. OS for grade 2 patients was 17 months versus 18 months in h-GBM patients. Panel B: overall survival curves of grade 2 patients within the m-GBM group; patients with an isolated *TERT*p mutation (so absence of *EGFR* amplification and/or 7+/10−) versus patients with an *EGFR* amplification and/or 7+/10− with or without a *TERT*p mutation (all but 2 of these patients also harbored a *TERT*p mutation). OS was 17 months for isolated *TERT*p mutation versus 21 months for patients with *EGFR* amplification and/or 7+/10−.

Out of the 81 histological grade 2 cases (original diagnostics) in the m-GBM cohort, upon pathology review 60 patients were still classified as grade 2 histology, in 15 patients the histology was revised into grade 3 as defined above, and in 6 patients into grade 4 as (small foci of) necrosis or vascular proliferation were detected upon reinvestigation. Out of the 21 histological grade 3 patients, 11 were again classified as grade 3 histology after revisions. Five patients were revised as grade 2 histology, and the other 5 as grade 4 for reasons listed above. Median age did not differ between groups (grade 2 histology: 61 years, grade 3 histology: 58 years, grade 4 histology: 64 years, h-GBM: 55 years). Clinical presentation of m-GBM was different compared to h-GBM. m-GBM lacking high-grade features more often presented with seizures than h-GBM (56% vs. 27%). Primary surgical strategy was also different with a lower percentage of resections (m-GBM: 20% vs. h-GBM: 80%) similar to our previous analysis of the smaller cohort.^4,5^ We found no significant OS difference between histological grades in our m-GBM cohort and no difference with h-GBM (Figure 1A). Additionally, in cases with grade 2 histology, we found no difference in OS between patients with an isolated *TERTp* mutation compared to cases with additional *EGFR* amplification and/or 7+/10− (Figure 1B).

To conclude, in our cohort of glioblastomas with grade 2 histology and imaging characteristics of low-grade glioma, we observed a different clinical presentation compared to histologically defined glioblastoma. However, we could not confirm the results of Berzero et al., where histological grade impacts OS and where isolated *TERT*p mutations are associated with longer OS.^3^ Although our series is larger and supports the concept of the proposed CNS5 classification, we feel more independent series with pathology reviews that investigate the course of GBMs lacking high-grade features are needed to fully answer this grade issue.
